# Comment on “Therapeutic Effects and Biomarkers in Sublingual Immunotherapy: A Review”

**DOI:** 10.1155/2012/969861

**Published:** 2012-07-05

**Authors:** George F. Kroker, Vijay K. Sabnis, Mary S. Morris, James C. Thompson

**Affiliations:** Allergy Associates of La Crosse, Ltd., Onalaska, WI 54650, USA

## Abstract

Numerous sublingual immunotherapy studies have shown efficacy using a wide variety of dosing regimens. Despite a few grade III and one anaphylactic reaction due to a patient over-dose, there have been no fatal reactions resulting from sublingual immunotherapy treatment. Although safer than SCIT, SLIT is still immunotherapy. Special consideration should be given to what will ensure the highest level of safety for the patient given his or her history, exam and allergy test results. Dosing levels for sublingual immunotherapy should be based on what is therapeutically effective for each individual patient and adjusted accordingly throughout the treatment course.

In the recent review article “Therapeutic effects and biomarkers in sublingual immunotherapy: a review,” it is stated “several case reports have also described anaphylactic shock or severe fatal reactions induced by sublingual [1–4] administration of allergens.” We reviewed the references included in the article and although there were systemic reactions, there are no case reports of fatal outcomes. The first reference speculated the reaction was due to dose-concentration allergen exposure during a high pollen season while using a high-dose protocol for sublingual immunotherapy [1]. The second reference included two cases of severe reactions from the standardized dosed tablet, Grazax. The first, a pediatric patient, was prescribed the tablet in addition to an existing SCIT regimen. Despite reactions using the same strength for moderately and strongly allergic patients, the authors remarkably suggest tablets are the safest approach for SLIT, especially in children. Sublingual immunotherapy with drops allows for increases or decreases of dosing dependent on the severity of allergy. In the second, an adult patient, it was unclear whether she was being cotreated with SCIT and SLIT. After attempted grass and birch SCIT, the patient was unable to tolerate both so SCIT for grass was discontinued. After the following year grass season, the patient began taking Grazax and did not take the first dose under the supervision of a physician. She had an immediate reaction suggesting the starting dose was too high for her severe allergies [2]. The third reference discussed two adolescent cases in which neither patient previously tolerated SCIT but was dosed using an ultrarush protocol resulting in grade III reactions [3]. The fourth reference did include a case of anaphylaxis; however, the individual discontinued her maintenance dose for three weeks and then continued taking SLIT at six times the prescribed dose, 60 drops at once [4].

The article also states later in the same paragraph “despite the few case reports of severe fatal events, life threatening severe fatal reactions have not been found in clinical trials.” To our knowledge, there have been no fatal events with sublingual immunotherapy. We respectfully request for you to correct the inaccurate statements that SLIT has caused severe fatal reactions. In medical parlance, fatal means death, and while the authors cite anaphylactic and grade III reactions to sublingual immunotherapy, there is no literature available to support the claim of “fatal.”

Although safer than SCIT, SLIT is still immunotherapy. Particular caution needs to be used for patients with prior systemic reactions to SCIT. Doses should be monitored and may need to be modified during treatment. First doses should be administered in the physician's office.

In our experience, multiantigen sublingual immunotherapy treatment that is dosed using the patient's allergen test results is both effective and maintains an excellent safety profile. Threshold dosing can be administered using a number of environmental allergens [5, 6]. We have observed a combination of clinical symptom improvements, reduced skin test reactivity, and decreases in specific IgE levels in our forty-year history in treating 125,000 patients from the United States.

## References

[1] Eifan AO, Keles S, Bahceciler NN, Barlan IB (2007). Anaphylaxis to multiple pollen allergen sublingual immunotherapy. *Allergy*.

[2] De Groot H, Bijl A (2009). Anaphylactic reaction after the first dose of sublingual immunotherapy with grass pollen tablet. *Allergy*.

[3] Cochard MM, Eigenmann PA (2009). Sublingual immunotherapy is not always a safe alternative to subcutaneous immunotherapy. *Journal of Allergy and Clinical Immunology*.

[4] Blazowski L (2008). Anaphylactic shock because of sublingual immunotherapy overdose during third year of maintenance dose. *Allergy*.

[5] (2008). *La Crosse Method; Sublingual Immunotherapy Practice Protocol*.

[6] Morris MS, Lowery A, Theodoropoulos DS, Duquette RD, Morris DL (2012). Quality of life improvements with sublingual immunotherapy: a prospective study of efficacy. *Journal of Allergy*.

